# PD-L1 Expression in 65 Conjunctival Melanomas and Its Association with Clinical Outcome

**DOI:** 10.3390/ijms21239147

**Published:** 2020-11-30

**Authors:** Sandra Lassalle, Sacha Nahon-Esteve, Eric Frouin, Camille Boulagnon-Rombi, Nicolas Josselin, Nathalie Cassoux, Raymond Barnhill, Boris Scheller, Stéphanie Baillif, Paul Hofman

**Affiliations:** 1 Laboratory of Clinical and Experimental Pathology, Centre Hospitalier Universitaire de Nice, University Côte d’Azur, Pasteur 1 Hospital, 30 avenue de la voie Romaine CS 51069, 06001 Nice CEDEX 1, France; lassalle.s@chu-nice.fr; 2 Institute of Research on Cancer and Aging of Nice (IRCAN), INSERM U1081/CNRS UMR7284, Medical School 28, Avenue de Valombrose, 06107 Nice CEDEX 2, France; 3 FHU OncoAge, Centre Hospitalier Universitaire de Nice, University Côte d’Azur, Pasteur Hospital, 30 avenue de la voie Romaine CS 51069, 06001 Nice CEDEX 1, France; 4 Hospital-Integrated Biobank (BB 0033-00025), Laboratory of Clinical and Experimental Pathology, Pasteur 1 Hospital, 30 avenue de la voie Romaine CS 51069, 06001 Nice CEDEX 1, France; 5 Department of Ophthalmology, Pasteur 2 Hospital, 30 avenue de la voie Romaine CS 51069, 06001 Nice CEDEX 1, France; nahon-esteve.s@chu-nice.fr (S.N.-E.); baillif.s@chu-nice.fr (S.B.); 6 Laboratory of Pathology, Centre Hospitalier Universitaire de Poitiers, 2 rue de la Milétrie, CS 90577, 86021 Poitiers CEDEX, France; Eric.FROUIN@chu-poitiers.fr; 7 Laboratory of Pathology, Centre Hospitalier Universitaire de Reims, avenue du Général Koenig, 51092 Reims CEDEX, France; cboulagnon-rombi@chu-reims.fr; 8 Institut d’Histo-Pathologie, 55 rue Amiral du Chaffault, CS 50424, 44104 Nantes CEDEX 4, France; njosselin@ihp-pathologie.fr; 9 Department of Ophthalmology, Institut Curie, 26 rue d’Ulm, 75248 Paris CEDEX 5, France; nathalie.cassoux@curie.fr; 10 Department of Pathology, Institut Curie, 26 rue d’Ulm, 75248 Paris CEDEX 5, France; raymond.barnhill@curie.fr; 11 Faculty of Medicine University of Paris Descartes, 15 rue de l’École de Médecine, 75006 Paris, France; 12 Department of Epidemiology and Biostatistics, CLCC CAL, 33 avenue de Valombrose, 06189 Nice CEDEX 2, France; boris.scheller@nice.unicancer.fr

**Keywords:** PD-L1, conjunctival melanoma, PD-1, CD8, prognosis

## Abstract

Conjunctival melanoma (CM) iss a rare and aggressive tumour that is increasing in frequency. The prognostic value of PD-L1 expression, alone or in combination with CD8 and PD-1 expression and the *BRAF* and *NRAS* status, has not been determined in CM to date. We evaluated the expression of PD-L1, CD8, PD-1 in CM and investigated whether there was an association between the expression of these markers and the *BRAF* and *NRAS* molecular profile as well as some clinico-pathological criteria. A total of sixty-five CM were assessed for PD-L1, PD-1, and CD8 expression by immunohistochemistry (IHC) and for *BRAF* and *NRAS* genomic alterations using molecular biology techniques and anti-BRAF and anti-NRAS antibodies. PD-L1 expression in tumour cells (TC) was very low or absent but detected in tumour-infiltrating immune cells (IC). A correlation was observed between the expression of PD-L1, CD8, and PD-1 in IC. No correlation between PD-L1 expression (in tumour and/or immune cells) and *BRAF* or *NRAS* mutations was observed. PD-L1 expression in IC correlated with a higher pTNM stage and PD-L1 expression in TC with worse disease-specific survival. PD-L1 expression is a potential prognostic biomarker that correlates with poor prognosis in CM patients.

## 1. Introduction

Ocular melanomas include uveal melanoma and conjunctival melanoma (CM). CM represent 5% of melanomas in the ocular region [1] and 0.25% of all melanomas [2]. CM are rare and aggressive tumours of the middle-aged and elderly, and the incidence is increasing [1,3]. CM is much more similar to cutaneous melanoma than to uveal melanoma at the molecular level, but also considering the clinical evolution. As for cutaneous melanoma, CM can be metastatic to all organs, while the most common metastatic site of uveal melanoma is the liver. Uveal and CM display distinct genetic features, which should be taken into consideration when making clinical decisions. Notably, genetic studies have reported mutations in *BRAF*, *NRAS*, and *KIT*, and implication of ultraviolet radiation as a risk factor for CM [4,5,6,7,8,9,10]. Indeed, large cohorts of CM patients have identified *BRAF* and *NRAS* mutations in 29–50% and in 18%, respectively [8,10,11]. Conversely, no *BRAF* or *NRAS* mutations or UV-induced mutational signatures have been described in uveal melanoma [12].

Programmed death-ligand-1 (PD-L1), an immune inhibitory protein mainly express but not only in tumour cells, binds to programmed death-1 (PD-1) expressed on the tumour-infiltrating lymphocytes, in order to suppress anti-cancer immunity and enable neoplastic growth [13]. In this context, checkpoint-blockade immunotherapy is one of the most promising advances in treatment of many solid tumours in several years, including for melanoma [14]. Therefore, immunotherapies have been successfully exploited in the treatment of metastases of cutaneous melanoma and have led to long-lasting clinical responses [15,16,17,18,19,20]. However, currently it is not certain whether assessment of PD-L1 expression on tumours is necessary to guide prediction of the treatment response to anti-PD-1 therapies. Moreover, the best first line treatment in the whole population of advanced melanoma patients as well as the best strategy to use in non-responders is not clearly defined [21,22]. Several cases of unresectable or metastatic CM treated with anti-PD-1/PD-L1 inhibitors were recently described [23,24,25,26]. Only two studies have already evaluated PD-L1 expression in CM while only one found expression of PD-L1 in a cohort of 27 CM [27,28].

There are no established prognostic and predictive markers for CM until now. Thus, there is an urgent need for a comprehensive evaluation in CM behaviour in combination with different biomarkers. The objective of this study was to evaluate PD-L1 expression in a multi-centre cohort of 65 primary CM, and to compare it with: (i) PD-1+ and CD8+ expression in intra-tumoral lymphocyte infiltrates, (ii) the *BRAF* and *NRAS* status, (iii) histopathological variables, and (iv) the clinical follow-up of patients.

## 2. Results

### 2.1. Immunohistochemical PD-L1 Expression

**Figure 1:**
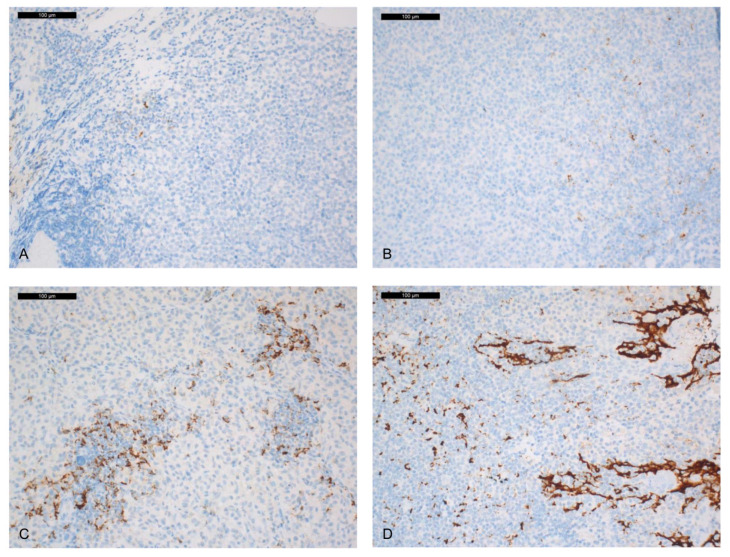
PD-L1 expression in immune cells with the SP142 clone (the panels with different percentages of PD-L1 expression in IC are representative of images showing similar results). (**A**) rare IC staining (1%). (**B**) focal IC staining (5%). (**C**) moderate IC staining (20%). (**D**) positive control (immuno-peroxidase; original magnification ×200).

A total of 64 out of 65 (98%) cases were analysed with the SP142 antibody (Ab) (one case could not be analysed due to over-pigmentation). No labelling of tumour cells (TC) was noted. Labelling of tumour-infiltrating immune cells (IC) was found in 28/64 (43.7%) cases (Figure 1).

**Figure 2:**
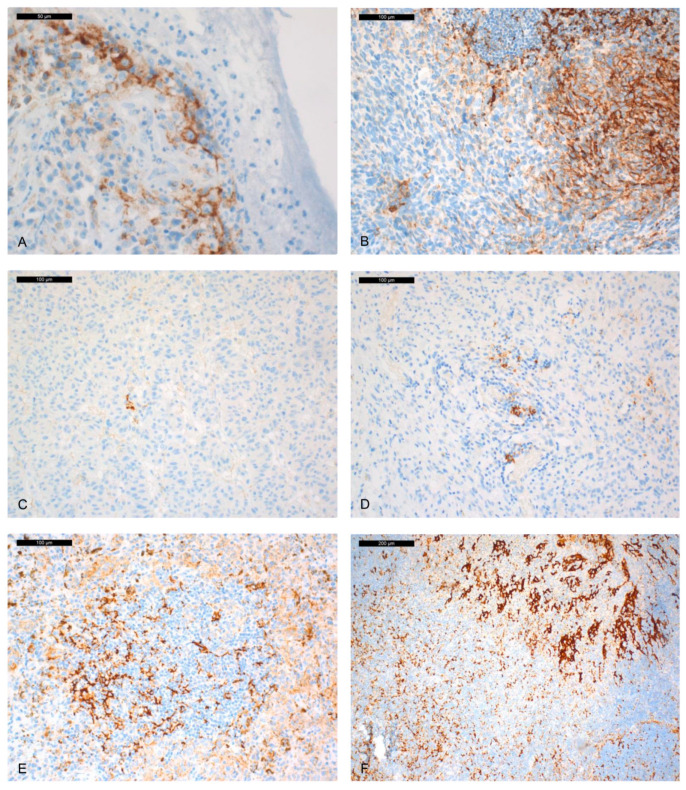
PD-L1 expression in tumour and immune cells with the SP263 clone (the panels with different percentages of PD-L1 expression in TC and IC are representative of images showing similar results). (**A**) staining of very few tumour cells (TC) (3% of all tumour cells). (**B**) focal TC staining (15% of all TC). (**C**) rare immune cell (IC) staining (1%). (**D**) focal IC staining (5%). (**E**) moderate IC staining (20%). (**F**) Positive control (immuno-peroxidase; **A**: ×400; **B**–**E**: ×200; **F**: ×100).

A total of 60 out of 65 (92%) cases were analysed with the SP263 Ab. A total of 5 out of the 65 tumours could not be analysed, because pigmentation interfered with the interpretation (1 case) or absence of residual tumour material (4 cases) following use of the paraffin blocks. Labelling of TC was observed in 6/60 (10%) cases (between 3% and 15% stained TC, depending on the tumours) (Figure 2). IC were PD-L1 positive in 35/60 (58.3%) cases (Table 1).

Using both PD-L1 clones, PD-L1-positive IC were mainly disposed as aggregates within the tumour, as well as aggregates towards the periphery of the tumour.

The inter-observer coefficient of agreement (Cohen Kappa) was performed to compare the expression of PD-L1 in IC with SP142 and SP263 Abs. A good correlation was observed for IC between the SP263 and SP142 Abs (0.74). This indicated that the IC+ cases were relatively concordant with both Abs. No concordance between TC was evaluated because of absence of TC stained with the SP142 Ab.

### 2.2. CD8^+^ PD-1 and NKP46 Tumour-Infiltrating Lymphocyte (TIL) Expression

A total of 5 out of the 65 (7%) tumours could not be analysed for CD8^+^ TILs (due to strong pigmentation or absence of residual tumour material). A total of 8 out of 60 (13.3%) cases had more than 50% CD8^+^ TILs, 26/60 (43.3%) cases between 5% and 50%, 20/60 (33.3%) cases less than 5% CD8^+^ TILs, and 6/60 (10%) cases showed no CD8^+^ TILs. Most of the CD8^+^ TILs were intra-tumoral, but for 3 cases (5%) that were located on the periphery of the tumour.

Just 1 tumour out of the 65 could not be analysed for PD-1^+^ TILs (strong pigmentation). Just 1 out of 64 (1.5%) cases had more than 50% TILs expressing PD-1, 14/64 (21.9%) cases between 5 and 50%, 10/64 (15.6%) cases less than 5%, and 32/64 (50%) cases no PD-1-expressing TILs.

A total of twenty-seven tumours could be analysed for NKP46 (few tumours left for analyse due to the very small size of biopsies). A total of 15 out of 27 were negative and 12 were positive (5 cases 1%, 3 cases 2%, 1 case 3%, and 3 cases 4%).

### 2.3. BRAFV600E and NRASQ61R Mutations

A total of 3 tumours out of 65 could not be analysed by immunohistochemistry (IHC) (strong pigmentation or absence of residual tumour material). Positive labelling for BRAFV600E for 8/62 (13%) cases and positive labelling for NRASQ61R in 4/62 (6%) cases was observed. Pyrosequencing for *BRAF* and *NRAS* mutations was performed for 30/65 (46%) cases, confirming detection of the BRAFV600E mutation by IHC in 8 cases, and the NRASQ61R mutation in 4 cases, without any discordant case. The mutations BRAFV600E and NRASQ61R were mutually exclusive.

### 2.4. Correlation between PD-L1 Expression and CD8/NKP46/PD-1^+^ TILs, BRAFV600E and NRASQ61R Mutations

A significant correlation between the number of CD8^+^ TILs and the number of PD-1^+^ TILs (*p* = 0.04) was observed. SP142/PD-L1-IC and SP263/PD-L1-IC positivity (≥1%) correlated with CD8^+^ TILs and with PD-1^+^ TILs (*p* = 0.04 and *p* < 0.001, respectively for SP142, and *p* = 0.0006 and *p* = 0.0002 for SP263, respectively). No correlation between the expression of PD-L1 in TC (SP263) and CD8^+^ TILs or PD-1^+^ TILs was observed. No correlation was found between PD-L1 expression (SP142 or SP263) or PD-1^+^ or CD8^+^ TILs and *BRAF* or *NRAS* mutations.

NKP46 expression (all positive staining versus negative) was significantly associated with PD-1 staining (*p* = 0.02). No other correlation was found for NKP46.

### 2.5. Association to Clinicopathological Parameters and Patient Outcome

A significant correlation was found between mitosis ≥1, recurrence, and worse disease free survival (DFS) (*p* = 0.0065 and 0.015 respectively). A non-limbal localization (bulbar non-limbal, caruncular, forniceal, and palpebral) was also poorly prognostic with worse DFS (*p* = 0.03). We did not find any significant prognostic correlation with gender, presence of epithelioid cells (epithelioid/mixed versus fusiform CM), tumour thickness (<2mm versus ≥2mm), presence of associated lesions (primary acquired melanosis (PAM)). The presence of a metastatic lymph node (pN1) or a distant metastasis (pM1) was associated with worse DFS (*p* = 0.07 (trend) and 0.02, respectively) and worse disease-specific survival (DSS) (*p* < 0.0001 and *p* = 0.004, respectively).

Correlation of the histological prognostic criteria, pathological-Tumour-Node-Metastasis (pTNM) staging and the number of CD8^+^ and PD-1 TILs is shown in Table 2. A significant correlation was found between stronger CD8 staining and a non-bulbar localization, the absence of epithelioid cells and a high pTNM stage (2 or 3 or 4) (*p* = 0.007, 0.02, and 0.014, respectively). No correlation was found for NKP46. We did not find any correlation between clinicopathological items and PD-1 staining. We did not find any correlation between CD8+ TILs or PD-1 TILs or NKP46 TILs and DSS or DFS.

SP263/PD-L1-IC+ and SP142/PD-L1-IC+ were preferentially found in the non-bulbar localization (*p* = 0.04 and 0.05, respectively). SP142/PD-L1-IC+ was also preferentially found at the higher pTNM stages (*p* = 0.027). We did not find any significant correlation between clinicopathological items and SP263/PD-L1-TC.

Finally, negative or weak staining of IC with SP142 and with CD8 was significantly associated with a pathological-Tumour 1 (pT1) stage and a bulbar localization.

**Figure 3:**
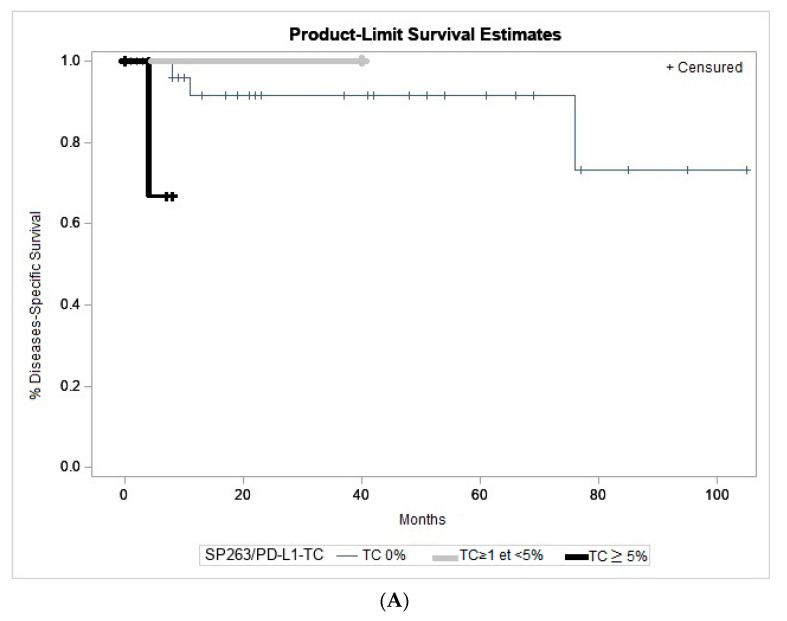

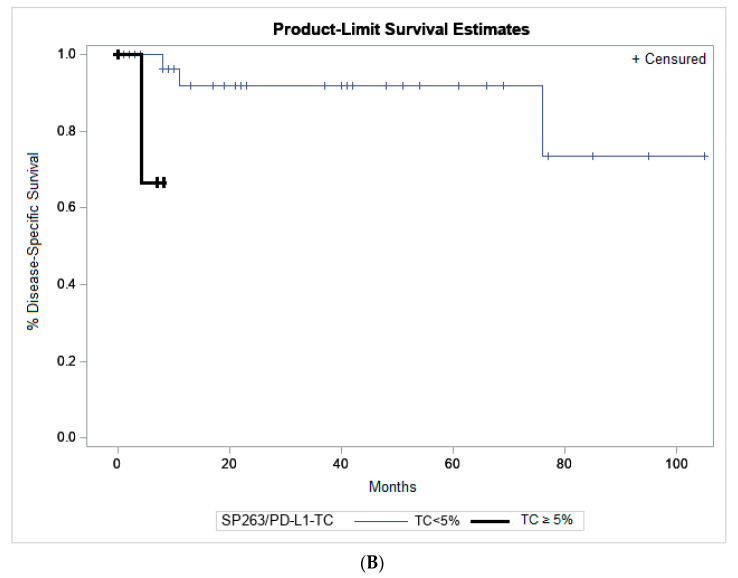
Kaplan–Meier analysis of disease-specific survival (melanoma-related death) according to the PD-L1 expression. SP263/PD-L1/TC+ was significantly associated with worse disease-specific survival in CM. (**A**) TC0 versus TC ≥1% and <5% versus TC ≥5% (*p* = 0.046). (**B**) TC0 and TC < 5% versus TC ≥ 5% (*p* = 0.013).

Melanoma TC staining was observed with only the SP263 clone. SP263/PD-L1-TC+ was poorly prognostic, with worse DSS (*p* = 0.046), reaching a *p* value of 0.013 if TC ≥ 5% (Figure 3A,B).

No significant association with DFS was noted with SP142 IC and SP263 IC or TC. No significant association with DSS was noted with SP142 and SP263 IC.

No significant association was found for *BRAFV600E* or *NRASQ61R* mutations and clinical or pathological parameters. There was no significant association of a *BRAF* mutation with DSS or DFS but a *NRAS* mutation was significantly associated with shorter DSS (*p* = 0.03).

Finally, a multifactorial analysis showed that metastasis (pM1) was independently associated with worse DFS (*p* = 0.01, hazard ratio = 5.54, IC95% OR (1.31–23.30)).

## 3. Discussion

Therapeutically blocking the PD-1/PD-L1 interaction may enhance antitumor immunity [13]. Detection of PD-L1 expression by IHC in patients with unresectable or metastatic cutaneous melanoma has been evaluated as a potential indicator of clinical efficacy for immune checkpoint inhibitors [22,29,30,31]. The purpose of our study was to analyse PD-L1 expression alone or in combination with CD8 and PD-1 expression and the BRAFV600E and NRASQ61R status, as a prognostic factor in CM. We tested 2 PD-L1 clones (SP142 and SP263) on 65 CM. It was recently shown that these two clones gave similar staining patterns with cutaneous melanomas [32]. However, the pattern of PD-L1 IHC in CM is not known with these two clones. Moreover, currently it is not certain that the epitopes recognized by these two clones are similar, both on TC and on IC. Finally, the administration of therapeutic molecules targeting PD1/PD-L1 for some solid tumours is linked to specific biomarkers used as FDA approved companion diagnostic tests (SP142 for atezolizumab and SP263 for pembrolizumab treatment) [33]. PD-L1 expression was mainly detected on IC with both clones, while only 10% of cases demonstrated PD-L1 expression in tumour cells with the SP263 Ab. The tumours with PD-L1/IC+ were practically the same with the SP142 and SP263 clones. Compared to other types of tumours, this preferential labelling of IC has already been reported in cutaneous melanoma and a correlation between the PD-L1 level of IC and response to treatment was found [34]. Only two studies evaluated PD-L1 expression in CM. IHC (clone E1L3N) has been used to investigate PD-L1 expression in 3 CM and did not show any labelling [27]. Immunofluorescence (IF) was used in another study of a cohort of 27 CM [28], in which PD-L1 (SP142 Ab) expression was detected in TC of 5 (19%) patients and in IC of 16 (59%) patients. We also found preferential labelling on IC, with clone SP142 (44%) and clone SP263 (58%). However, only clone SP263 showed TC staining in 6 CM (10%) in 3–15% of TC. However, currently, the IF technique is not the standard for PD-L1 detection in clinical routine practice, whereas IHC is the most widely used approach across studies, including those evaluating companion diagnostic tests [21,35].

Expression of PD-L1 alone may not be sufficient to identify the patients who respond to anti-PD-L1/PD-1 treatment. In particular, there is now considerable evidence for this in cutaneous melanoma patients [36,37,38]. Response may be linked to a high level of CD8+ and PD-1+ TILs [36,37,39], and other parameters [21,40,41]. We found a significant correlation between the positivity to PD-L1 (SP142 and SP263 clones) and CD8+ TILs and between the positivity of PD-L1 (SP142 and SP263 clones) and PD-1+ TILs. This correlation has also been reported for cutaneous melanoma [42,43]. In addition, it has been shown that an absence of response to anti-PD-L1/PD-1 treatment correlated with decreased expression of PD-L1 by IC or the absence of CD8+ T cell in the tumour mass prior to treatment [37,44]. Thus, the expression of PD-L1 and/or CD8 and/or PD-1 could potentially be useful in the selection of patients with CM who respond to anti-PD-L1/PD-1 therapy. However, studies looking for a correlation between expression and outcome in patients receiving these therapies are needed in the future. We were able to analyse NKP46 expression on 27 tumours. NKp46 is an important NK activating receptor shown to participate in recognition and activation of NK cells against tumour cells [45]. We intend to explore if NKP46 could be an interesting marker in CM, but no correlation was found between NKP46 and histological, immunohistochemical, and clinical parameters, except a significative correlation of NKP46 with PD-1 expression.

In cutaneous melanoma, the relationship between PD-L1 staining and prognosis observed across studies is still inconsistent [29,30,31,46,47,48]. However, several studies have reported similar association between PD-L1 expression and a poor prognosis in cutaneous melanoma [31,47,49,50] and other solid tumours [47]. Cao et al. showed that PD-L1 positive staining in the tumour was associated with worse CM-related survival [28]. In our study, CM with SP263/PD-L1/TC ≥ 5% was associated with shorter DSS (*p* = 0.0135) (Figure 3). No correlation with DFS was found. Therefore, expression of PD-L1 in tumour cells could be a new histopathological prognostic criteria in addition to localization, tumour thickness, recurrence, or the pTNM stage [51,52,53,54,55,56]. One of the limitations of our study is the lack of data such as the quality of the edges of the resected tumour or treatment with adjuvant radiotherapy. This could partially explain the high rate of recurrence observed in our cohort. This information is important for reliable evaluation of DFS and DSS. Therefore, further assessment of PD-L1 expression as a predictive biomarker in CM is still required.

In addition to immunotherapy, patients with melanoma can also benefit from anti-BRAF therapy when a mutation is detected [57,58]. We found 13% of BRAFV600E CM. This is a little bit lower than the data reported in the literature (18–50% of BRAF positive CM) when using different molecular techniques and for different cohorts [10,59,60]. This discrepancy could be explained in part by the fact that only 46% of the CM could be tested using pyrosequencing due to the samples size and to the tissue availability to make both immunohistochemistry and molecular biology approaches for each sample. Moreover, some *BRAF* and *NRAS* mutations could be missed by using IHC alone since the anti-BRAF and anti-NRAS antibodies allow detecting only some specific *BRAF* and *NRAS* mutations. Studies have reported benefit from association of anti-BRAF and anti-PD-L1/PD-1 treatment [61]. While no significant correlation between expression of PD-L1 and BRAFV600E was found in our cohort, five patients showed the BRAFV600E mutation and PD-L1+ on IC, which may suggest that a subgroup of patients may benefit from a combination of anti-BRAF and anti-PD-1/PD-L1 treatment.

A limitation of the present study is the small number of patients in some pT categories, since 72.3% of the tumours were staged pT1, and only one pT4 tumour. However, the observed prevalence of the pT1 stages reflected the daily practice of the different clinical centres participating in this retrospective study (no criteria for case selection).

## 4. Materials and Methods

### 4.1. Patient Cohort

The study was performed in accordance to the guideline of the declaration of Helsinki, and approved by the local research ethics committee (DRCI; CHUN, N° IE-2016-918; 15 November, 2016). Patient samples were retrieved from 8 French institutions: the Laboratory of Clinical and Experimental Pathology/Hospital-integrated Biobank (BB-0033-00025, Pasteur Hospital, Nice, France), the Pathology Departments of CHU of Poitiers (Poitiers, France), Brest (Brest, France), Reims (Reims, France), Creteil Hospital (Creteil, France), Institut Curie, (Paris, France), the IHP Laboratory (Nantes, France), and the DIAG Laboratory (Nice, France). Data transfer and use for statistical analyses were done in an anonymised manner. The clinical and pathological features of the CM are shown in Table 3. All cases were included retrospectively from January 2002 to January 2016. The CM were non bulbar in 12 patients and bulbar in 53 patients (28 bulbar limbal, 8 bulbar non limbal and 17 bulbar indeterminate). Tumour thickness was <2 mm in 40 cases and ≥2 mm in 25 cases (median thickness: 2.3 mm, range: 0.1–20). In clinical follow-up (median 29.2 months, range 0–105), local recurrence was observed in 17 patients, metastasis in 7 patients (2 liver, 1 sinusal, 1 uterine corpus, 1 lymph node, 1 osseous metastasis, and 1 not specified) and 9 patients died (4 related to the CM, 3 not related, and 2 with unknown cause).

A total of sixty-five patients with primary invasive CM were included. Histological sections were reviewed by 4 senior pathologists (SL, MI, VH, and PH). The clinico-pathological prognostic features of conjunctival melanoma were noted (localization, thickness, presence of epithelioid cells, associated lesion, mitotic rate, pTNM stage). Patients received information concerning the study and signed informed consent was obtained. The study was approved by the Ethics Committee of the University Côte d’Azur (Nice, France) and performed according to the guidelines of the Declaration of Helsinki (DC-2015-2454 (1 January 2016).

### 4.2. Immunohistochemistry

IHC was performed using a BenchMark ULTRA automated staining instrument (Ventana Medical Systems, Tucson, AZ, USA). Formalin-Fixed Paraffin Embedded (FFPE) freshly cut serial tissue sections of 3µm thickness, mounted on positively charged slides were stained for PD-L1 with two anti-human PD-L1 rabbit monoclonal Ab, according to the manufacturer’s recommendations [44]: SP263 and SP142 antibodies (Abs) (Ventana, Tucson, AZ, USA). Other sections were incubated with a rabbit monoclonal anti-BRAF Ab (clone VE1, Ventana, Tucson, AZ, USA) during 36 mn at 37 °C. The OptiView DAB IHC Detection Kit (Ventana, Tucson, AZ, USA) and the OptiView Amplification Kit (Ventana, Tucson, AZ, USA), were used according to the manufacturer’s recommendations [7]. Sections were counter-stained with hematoxylin and bluing reagent. Serial tissue sections were incubated with a mouse monoclonal anti-PD-1 Ab (clone NAT 105, Cell Marque, Rocklin, CA, USA) for 16 min at 37 °C, with a rabbit monoclonal anti-CD8 Ab (clone SP57; Ventana, Tucson, AZ, USA) for 20 min at 37 °C, with a rabbit monoclonal anti-NRAS Ab (Q61R) (clone SP174, Spring Bioscience, Pleasanton, CA, USA) for 32 min at 37 °C and with a mouse monoclonal anti-NKP46 (clone 195314, R&D Systems, Minneapolis, MN, USA) for 32 min at 37 °C. The UltraView IHC Detection Kit (Ventana, Tucson, AZ, USA) were used according to the manufacturer’s recommendations Each IHC run contained a positive control (tonsil for PD-L1, PD-1, CD8 and NKP46 and positive melanoma controls for BRAF and NRAS) and a negative Ab control (buffer, no primary Ab). In addition, 40 primary cutaneous melanomas were used as control as well as 20 lung adenocarcinomas known to express PD-L1 in more than 50% tumour cells.

### 4.3. Staining Evaluation

We evaluated PD-L1 expression based on scoring algorithms described in clinical trials using the corresponding anti-PD-L1 inhibitors

(1) PD-L1 SP142 staining was assessed on both TC and IC, as previously described according to the POPLAR scoring system [44]. SP142 was considered positive when ≥1% of TC (membranous staining) or ≥1% of IC (cytoplasmic dot-like signals) stained for PD-L1. All types of ICs were counted together.

(2) PD-L1 SP263 staining was considered positive when ≥1% of TC (membranous staining) or IC (cytoplasmic dot-like signals) stained for PD-L1 [62,63]. For statistical analysis, 1, 5, and 50%, and 1, 5, and 10% thresholds were used for TC and IC, respectively.

(3) TILs were evaluated on haematoxylin and eosin sections [64]. PD-1 staining was assessed on TILs and scored as focal, moderate or marked, as previously described [65]. CD8^+^ TILs staining and NKP46 staining were scored as focal (isolated, <5% of TILs), moderate (5–50%) and severe (>50%). NRASQ61R and BRAFV600E IHC expression was evaluated as previously described [66,67].

The IHC staining was independently assessed by 4 senior pathologists (SL, MI, VH and PH). When a discrepancy between the results was noted, the slides were jointly reviewed with a multi-head microscope to obtain a consensus.

### 4.4. BRAF and NRAS Molecular Analysis

The *BRAF* and *NRAS* mutational status was determined for 30/65 (46%) cases. Tumour DNA was isolated from FFPE tissue samples using the QIAamp DNA FFPE tissue kit (Qiagen, Hilden, Germany), according to the manufacturer’s instructions. Pyrosequencing of *NRAS* exon 1 (codon 12 and 13) and exon 2 (codons 60 and 61) using the Therascreen *NRAS* Pyro kit (Qiagen, Hilden, Germany) and *BRAF* exon 15 using the Therascreen *BRAF* Pyro Kit (Qiagen, Hilden, Germany), was performed as previously described

### 4.5. Statistical Analysis

The concordance between the analysed markers was determined with the χ2 test for recurrence and the mitotic rate or the Fisher test for associated lesions, primary site and pTNM (7th Ed.). The nonparametric Wilcoxon test was performed for thickness and age. The impact of the following factors: patient age, gender, localization, *NRAS* status, *BRAF* status, associated lesion, histological type, thickness, mitosis, pTNM stage, and immunohistochemical markers SP142 IC and TC, SP263 IC and TC, PD-1 TILs, CD8+ TILs, NKP46 TILs were investigated by univariate and multivariate analysis. Statistical analyses were performed using log rank tests, for univariate survival analysis. All variables associated with *p* < 0.10 on univariate analyses were included in Cox regression models. All statistical tests were performed with the R.3.4.3 software program for Windows, with a threshold significance of 5%. Kaplan–Meier survival curves were determined to assess the prognostic significance for DFS and DSS. DSS was defined as the interval between the date of surgery and the date of death (melanoma-related death only) or of the last follow-up. DFS was defined as the interval between the date of surgery and the date of the first relapse of the disease (local or metastatic) or of the last follow-up or of the date of death (melanoma-related death only). All statistical analyses and data presentations were performed with SAS Guide (version 5.1; SAS Campus Drive, Cary, USA). *p*-values < 0.05 indicated statistical significance. The Cohen’s kappa statistic was used to measure agreement between the 4 PD-L1 immunostainings (with IC positive = IC > 0 and TC positive = TC > 0). A kappa of 1 indicates perfect agreement whereas a kappa of 0 indicates agreement equivalent to chance.

## 5. Conclusions

In conclusion, we report PD-L1 IHC expression preferentially on IC in a large cohort of 65 CM. Our results show that PD-L1 expression in tumour cells is a potential prognostic indicator for poor outcome in CM patients. Correlation between PD-L1 IHC expression and an objective response to anti-PD-1/PD-L1 treatment needs to be investigated. If validated in the near future by independent studies that the response to immune checkpoint inhibitors is indeed related to the expression of immunohistochemical markers (such as PD-L1 and/or CD8 and/or PD1) in CM, then looking for the expression of the biomarkers could potentially be of interest in the selection of patients for anti-PD-L1/PD-1 therapy. Therefore, it could be mandatory in the future to ask for the PD-L1, CD8, and PD1 status in CM and according to these different protein expressions to consider an ICI treatment.

## Figures and Tables

**Table 1:** PD-L1 TC and IC staining with the 2 PD-L1 antibodies.

PD-L1 IHC Assays	Not Determined (Insufficient Material, Melanin)	PD-L1 Expression in Tumour Cells Nb. of Cases (%) (Range)	PD-L1 Expression in Immune Cells IC<1 Nb. of Cases (%)	PD-L1 Expression in Immune Cells IC≥1% and <5% Nb. of Cases (%)	PD-L1 Expression in Immune Cells IC≥5% and <10% Nb. of Cases (%)	PD-L1 Expression in Immune Cells IC≥10% Nb. of Cases (%)
SP142 (*n* = 64)	1	0 (0%)	36 (56%)	5 (8%)	11 (17%)	12 (19%)
SP263 (*n* = 60)	5	6 (10%) (3–15% TC)	25 (42%)	5 (8%)	9 (15%)	21 (35%)

**Table 2:** Correlation between the criteria for histological prognosis and the number of CD8^+^ and PD-1 TILs.

Clinical and Pathological Features	CD8					PD-1				
	0	<5%	≥5% and ≤50%	>50%	*p* Value	0	<5%	≥5% and ≤50%	>50%	*p* Value
**PAM associated melanoma**										
Yes	0	2	1	0	0.7	25	7	9	0	1
No (de novo melanoma)	2	13	20	3		2	0	1	0	
**Presence of epithelioid tumour cells**										
Yes	6	19	25	4	0.02	35	9	13	1	1
No	0	1	1	3		4	1	1	0	
**Primary site**										
Limbal	19	5	2	0	0.14	1	7	13	1	0.058
Non-limbal	10	2	7	1		2	7	6	5	
Bulbar	6	16	23	2	0.007	34	9	8	0	0.3
Non-bulbar	0	4	3	5		5	1	5	1	
**Thickness**										
<2mm	2	10	19	4	0.21	24	6	8	0	0.79
≥2mm	4	10	7	3		15	4	5	1	
**Mitotic rate/mm^2^**										
<1	1	10	12	2	0.4	15	5	7	0	0.7
≥1	5	10	14	5		24	5	6	1	
**pTNM**										
pT1	3	15	22	2	0.014	30	8	8	0	0.06
pT2-3-4	1	2	4	5		6	1	3	2	
**Recurrence**										
Yes	3	5	8	0	0.3	11	2	3	0	0.6
No	2	7	10	4		15	6	6	0	
**Metastasis**										
Yes	0	4	2	0	0.08	4	1	1	0	1
No	4	4	14	4		18	5	7	0	

pTNM according the 7th UICC edition.

**Table 3:** Clinical and pathological features of conjunctival melanomas.

Clinical and Pathological Features	Conjunctival Melanomas *n* = 65 (%)	Recurrence *n* (%)	Metastases *n* (%)	Melanoma-Related Death *n* (%)
Age at diagnosis, median (range), y	69.4 (28–95)	71.2 (47–90)	64 (49–77)	68.2 (56–77)
**Gender**				
Male	25 (38)	12 (70%)	2 (29%)	2 (50%)
Female	40 (62)	5 (30%)	5 (71%)	2 (50%)
**Associated lesion**				
PAM	43 (66)	11 (92%)	7 (100%)	4 (100%)
Nevi	0 (0)	0	0	0
None (de novo)	7 (11)	1 (8%)	0	0
Recurrence	6 (9)	0	0	0
**Histotype**				
Epithelioid	44 (68)	14 (82%)	6 (86%)	4 (100%)
Fusiform	5 (8)	0	0	0
Mixed	16 (24)	3 (18%)	1 (14%)	0
**Primary site**				
Bulbar	53 (81)	13 (76%)	5 (71%)	3 (75%)
Bulbar limbal	28 (53)	5 (38)	2	1 (33)
Bulbar non limbal	8 (15)	4 (31)	2	2 (67)
Bulbar unknown	17 (32)	4 (31)	1	0
Palpebral	5 (8)	2 (12%)	1 (14%)	0
Forniceal	5 (8)	2 (12%)	1 (14%)	1 (25%)
Caruncular	2 (3)	0	0	0
**Thickness**				
<2mm	40	9 (53%)	3 (43%)	2 (50%)
≥2mm	25	8 (47%)	4 (57%)	2 (50%)
Mitotic rate/mm^2^				
<1	29 (45)	5 (29%)	3 (43%)	1 (25%)
≥1	36 (55)	12 (71%)	4 (57%)	3 (75%)
**pTNM**				
pT1a	10 (15)	1 (8%)	0	1 (33%)
pT1b	20 (31)	3 (25%)	1 (17%)	2 (66%)
pT1c	17 (26)	5 (42%)	3 (50%)	0
pT2a	1 (2)	0	0	0
pT2b	2 (3)	1 (8%)	0	0
pT2c	6 (9)	1 (8%)	1 (17%)	0
pT3	2 (3)	1 (8%)	1 (17%)	0
pT4	1 (1)	0	0	0

Associated lesion means pre-existing lesion). Recurrence: melanoma recurrence secondary to previous surgical resection of the tumour. Abbreviations: PAM: Primary Acquired Melanosis. pTNM according to the 7th Union for International Cancer Control (UICC) edition.
